# Engineered decellularized tendon hydrogel with sustained zinc ion release orchestrates anti-inflammatory microenvironment and functional regeneration in Achilles tendinopathy

**DOI:** 10.1016/j.mtbio.2025.102104

**Published:** 2025-07-16

**Authors:** Xiang Gao, Senyi Wu, Zheyu Yao, Yiding Shao, Jiahui Feng, Zheyang Yuan, Haijiao Mao

**Affiliations:** Department of Orthopaedic Surgery, The First Affiliated Hospital of Ningbo University, Ningbo, Zhejiang, 315010, China

**Keywords:** Achilles tendinopathy, Zinc oxide nanoparticles, Decellularized porcine Achilles tendon hydrogel, Inflammation

## Abstract

Achilles tendinopathy (AT), a prevalent degenerative tendon pathology characterized by dysregulated inflammatory pathways and compromised tissue healing capacity, necessitates the development of novel therapeutic approaches targeting molecular mechanisms of tendon repair. This study developed a zinc oxide nanoparticle (ZnO NPs)-integrated decellularized Achilles tendon extracellular matrix (DAT) hydrogel (Z-D) for minimally invasive AT treatment. The composite hydrogel exhibits thermosensitivity and enhanced mechanical stability through Zn^2+^-mediated crosslinking. *In vitro* biocompatibility assessments revealed that 0.25 % Z-D significantly promoted the proliferation capacity of Tendon-Derived Stem Cells (TDSCs), while simultaneously suppressing the expression of key pro-inflammatory mediators, such as interleukin-6 (IL-6) and tumor necrosis factor-alpha (TNF-α). Under Z-D treatment, TDSCs exhibited accelerated migration and upregulated tenogenic differentiation markers (scleraxis: SCX; tenomodulin: TNMD). In a rat AT model, Z-D implantation achieved multi-level repair: biomechanical restoration and collagen remodeling recovery. Mechanistically, Zn^2+^ release concurrently promoted TDSCs differentiation and inflammation resolution. Histological evaluation confirmed tendon structural recovery with minimal adhesion formation, and systematic biosafety assessment revealed no organ toxicity. This dual-functional hydrogel system establishes a paradigm for microenvironment-modulating AT therapy by orchestrating anti-inflammatory signaling and regenerative extracellular matrix reconstruction.

## Graphical abstract

Illustration of Z-D hydrogel's dual functions in modulating the inflammatory microenvironment and facilitating tendon regeneration. The released ZnO from Z-D hydrogel attenuate inflammation through inhibition of IL-6 and TNF-α expression, while promoting tendon repair by upregulating COL I, TNMD, and SCX expression alongside downregulation of COL III. (SCX: scleraxis; TNMD: tenomodulin; COL I: type I collagen; COL III: type III collagen; IL-6: interleukin-6; TNF-α: tumor necrosis factor-α).

**Figure:**
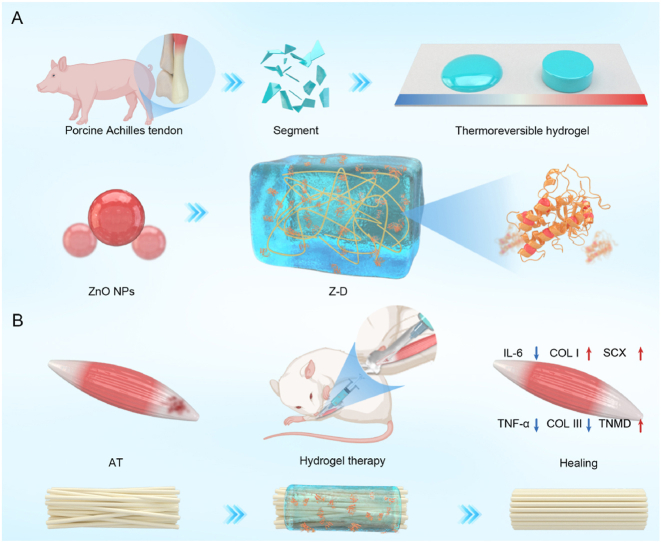

## 1 Introduction

Achilles tendinopathy (AT), a clinically prevalent degenerative tendon disease [1], has exhibited a marked global increase in both incidence and disability rates Epidemiological investigations reveal that degenerative tendinopathy affects 23.9 % of athletic populations, significantly higher than the 5.9 % prevalence in general populations, with adult population incidence approximating 2–3 cases per 1000 individuals [2]. The pathological progression of this disease primarily involves the synergistic effects of mechanical stress accumulation and age-related degeneration, clinically manifesting as characteristic localized pain, swelling, and motor dysfunction [[3], [4], [5]]. Histological analyses reveal that typical pathological features of AT include disordered collagen fiber architecture, abnormal cellular proliferation, and a chronic low-grade inflammatory microenvironment, which collectively lead to progressive deterioration of tendon biomechanical properties and eventual progression to Achilles tendon rupture [6,7].

Current clinical conservative strategies for AT mainly include exercise rehabilitation therapy [8] physical interventions (ultrasound therapy) [9], glucocorticoids, non-steroidal anti-inflammatory drugs (NSAIDs) and platelet-rich plasma (PRP) local injections [10]. However, existing therapies are largely limited to symptomatic relief, with minimal efficacy in reversing degenerative pathology. This underscores the need to develop novel therapeutic strategies with targeted repair capabilities from the perspective of pathological microenvironment modulation.

Recent studies highlight the critical role of immune microenvironment imbalance in AT pathogenesis. Mechanical overload triggers cascading responses by inducing collagen damage, tenocyte apoptosis, and NLRP3 inflammasome activation (the expression of pro-inflammatory mediators, particularly IL-1β) [11]. This signaling cascade induces persistent secretion of pro-inflammatory cytokines, notably TNF-α and IL-6 [12], forming a chronic inflammatory microenvironment through immune-matrix cell interactions, resulting in extracellular matrix (ECM) metabolic imbalance, aberrant tenocyte differentiation [13], and impaired regenerative repair. Therefore, modulating inflammatory responses may represent a pivotal breakthrough for reversing AT pathology [14].

Metal nanoparticle (1–100 nm)-based drug delivery systems offer novel insights for precision therapy of inflammatory diseases [15]. Through size control and surface functionalization, targeted drug delivery and controlled release can be achieved [16], among common metallic nanomaterials, gold nanoparticles (Au NPs) exhibit good biocompatibility and photothermal effects but lack inherent anti-inflammatory activity and face cost limitations [17]; Silver nanoparticles (Ag NPs) demonstrate notable antibacterial and matrix metalloproteinase inhibitory capacities [18], yet their cytotoxicity and stem cell differentiation inhibitory effects restrict applications [19]; Titanium dioxide nanoparticles (TiO_2_ NPs) enhance material mechanical properties and possess photocatalytic activity but require ultraviolet activation and may induce inflammatory responses [20]. In contrast, ZnO NPs exhibit unique advantages, Their anti-inflammatory activity is mediated through suppression of IL-6 and TNF-α expression in inflammatory pathways [[21], [22], [23]]. Notably, as an essential trace element, Zn^2+^ directly participates in cell cycle regulation and apoptotic signaling pathways, aligning closely with biological repair processes [24,25]. Although ZnO NPs have shown progress in bone regeneration, their anti-inflammatory-regenerative synergistic mechanisms and impacts in tendon repair remain underexplored [26].

Injectable hydrogels, as localized drug delivery carriers, hold significant potential for AT treatment. Naturally derived hydrogels (collagen, chitosan, hyaluronic acid) attract attention due to their excellent biocompatibility and biodegradability [[27], [28], [29], [30], [31]] Our previous studies demonstrated that porcine small intestinal submucosa (SIS) hydrogel (containing 90 % collagen) promotes cell recruitment and tissue remodeling through bioactive component release [32]. However, The decellularized porcine Achilles tendon matrix (DAT) hydrogel exhibits superior characteristics due to tissue specificity compared to xenogeneic ECM, DAT not only retains injectability and biocompatibility but also provides tendon-specific bioactive components (type I collagen) [[33], [34]]. Building on this, we innovatively developed a ZnO NP-loaded DAT composite hydrogel (Z-D) to synergistically integrate nanomaterial anti-inflammatory properties with tissue-specific ECM regenerative functions.

**Scheme 1:**
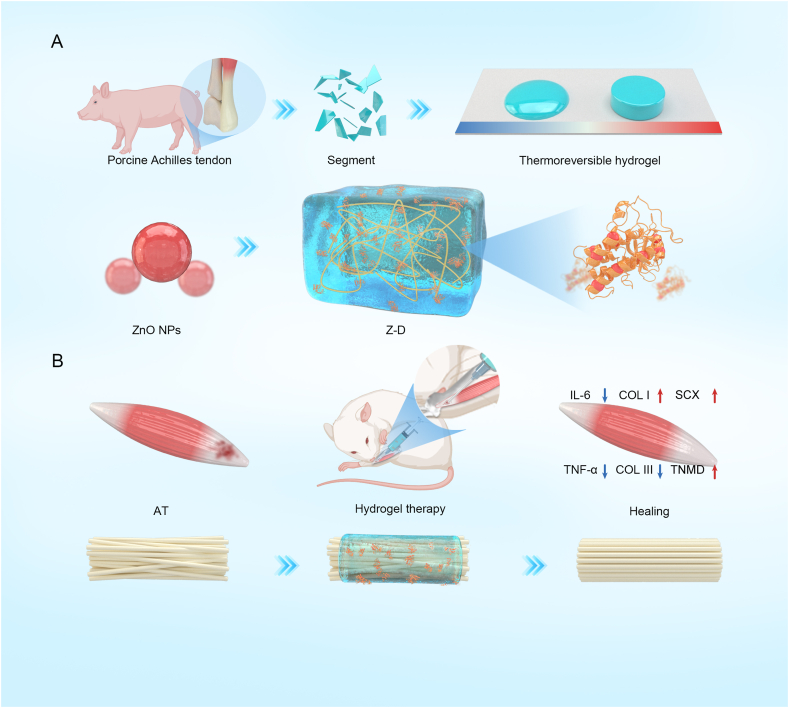
Schematic illustration of Z-D hydrogel's dual functions in modulating the inflammatory microenvironment and facilitating tendon regeneration. The released ZnO NPs from Z-D hydrogel attenuate inflammation through inhibition of IL-6 and TNF-α expression, while promoting tendon repair by upregulating SCX, TNMD, and COL I expression alongside downregulation of COL III.(SCX:scleraxis; TNMD: tenomodulin; COL I:type I collagen; COL III: type III collagen; IL-6: interleukin-6; TNF-α: tumor necrosis factor-α).

This study developed a novel Z-D hydrogel system for minimally invasive AT treatment. The hydrogel demonstrates excellent injectability, thermosensitivity, and moldability, enabling precise lesion filling via minimally invasive delivery. Its three-dimensional network structure effectively encapsulates pathological regions, reducing adhesion risks while achieving sustained ZnO NPs release. We systematically characterized the physicochemical properties of Z-D hydrogel and evaluated its effects on TDSCs tenogenic differentiation, inflammation-related gene and protein expression, and stem cell protection during Achilles tendon healing. Finally, using a rat AT model, we comprehensively assessed its *in vivo* therapeutic efficacy across histological repair, biomechanical performance, and molecular mechanisms (Scheme 1). Thus, this composite hydrogel is envisioned as a delivery system capable of seamlessly integrating material properties with biotherapeutic agents, offering valuable contributions to future drug delivery strategy development.

## 2 Materials and methods

### 2.1 Preparation of decellularized porcine Achilles tendon matrix hydrogel (DAT)

The decellularized porcine Achilles tendon Matrix hydrogel (DAT) was synthesized following established protocols with critical modifications as described below [[35], [36], [37]] In brief, freshly isolated porcine Achilles tendons were flash-frozen in liquid nitrogen for 5 min to facilitate mechanical fragmentation into 1 cm × 1 cm segments using sterile surgical scissors. To achieve complete decellularization, tissue fragments underwent two cycles of enzymatic digestion: each cycle involved 12 h incubation in 0.05 % trypsin-EDTA solution (4 °C, continuous agitation), followed by three sequential PBS washes(pH 7.4, 10 min per wash). Residual cellular debris was further removed through two 4h treatments with 1 % (w/v) sodium dodecyl sulfate (SDS) under gentle agitation. Post-decellularization, the tissue was sterilized via immersion in 75 % ethanol (20 min), lyophilized (FreeZone Triad, Labconco), and stored at −80 °C until decellularization efficacy validation (DNA quantification, histological analysis). For hydrogel fabrication, the lyophilized DAT was cryogenically milled into a fine powder and solubilized in 0.02 M HCl with 1 mg/ml porcine pepsin (Sigma-Aldrich, ≥2500 u/mg) under constant stirring at 4 °C for 48 h. The acidic suspension was neutralized to a physiological pH of 7.4 using 0.02 M NaOH, and the ionic strength was adjusted by adding 10 × PBS at a 1:10 (v/v) ratio. The pre-gel solution was maintained on ice to prevent premature gelation, then transferred to 37 °C for 1h to induce thermoresponsive hydrogel formation. Hydrogels with graded mechanical stiffness were prepared at final concentrations of 5, 10, 15, and 20 mg/ml. To engineer functional composites, zinc oxide nanoparticles (ZnO NPs, Sigma-Aldrich, <100 nm diameter) were homogenously dispersed into the DAT hydrogel at weight/volume ratios of 0.1 %, 0.25 %, 0.5 %, and 1 % [38],All composite hydrogels were aseptically aliquoted and subjected to immediate physicochemical characterization.

### 2.2 Assessment and characterization of decellularization efficiency in matrix hydrogel

To evaluate the decellularization efficacy of Achilles tendons: DNA quantification, hematoxylin-eosin (HE) staining, and collagen immunofluorescence analysis were conducted. Randomly selected Achilles tendon specimens from both pre-decellularization(pre-DAT)and post-decellularization(DAT)groups underwent DNA quantitative analysis (n = 3). DNA content was quantified using the CyQUANT Cell Proliferation Assay Kit (C7026, Invitrogen, China) following the manufacturer's protocol. HE staining was performed with an HE Staining Kit (Solarbio) following standard procedures. Tissue sections were processed through deparaffinization, primary antibody incubation, fluorescent-conjugated secondary antibody treatment, and nuclear counterstaining with DAPI (Solarbio).

For material characterization, scanning electron microscopy imaging(SEM) and X-ray photoelectron spectroscopy (XPS) analysis of DAT hydrogel and Z-D hydrogel were conducted using ZEISS GeminiSEM 360 (Germany) and Thermo Scientific K-Alpha (USA) instruments, respectively. The hydrogels were initially subjected to complete lyophilization in a freeze dryer. The freeze-dried specimens were then examined for morphological features using SEM. Fourier transform infrared spectroscopy (FTIR) analysis was conducted using the Thermo Fisher Scientific Nicolet iS20 spectrometer (USA). while Transmission electron microscopy (TEM) analysis was conducted on a JEOL JEM-F200 instrument (Japan).

### 2.3 *In vitro* degradation of the hydrogels

To investigate the degradation characteristics of hydrogels, freeze-dried hydrogel samples with an initial mass of 0.1 g were immersed in 10 mL PBS buffer containing 0.01 mg/mL Type I collagenase. The enzymatic degradation process was carried out in an oscillating incubator maintained at a constant temperature of 37 °C,with the buffer solution being replaced every 48 h to maintain enzymatic activity. Upon reaching predetermined time intervals, samples were retrieved and thoroughly rinsed with ultrapure water five times to remove enzymatic byproducts and residual salts, followed by lyophilization treatment. The mass loss percentage was calculated using formula: Mass loss (%) = (W_0_ - W_1_)/W_0_ × 100 %, where W_0_ represents the initial mass and W_1_ denotes the post-degradation mass. Each experimental group consisted of three independent replicates. This experimental protocol enables us to determine the mass loss of hydrogels during the degradation process, thereby gaining deeper insights into the time-dependent degradation behavior of hydrogels.

### 2.4 *In vitro* ion release of hydrogels

The *in vitro* ion release was measured by immersing 1 ml hydrogel containing 0.25 % ZnO NPs in 10 ml of PBS (37 °C) for different durations; at predetermined time points, the solution was collected and replaced with an equal volume of fresh PBS, and the concentration of zinc ions released was quantified using Inductively Coupled Plasma Atomic Emission Spectrometry (ICP-AES, Prodigy Plus, Teledyne Leeman Laboratories, USA).

### 2.5 Mechanical property test of hydrogel compression

The compressive properties of the hydrogels were tested using an electromechanical testing system(Model #3366, Instron Corporation, Shanghai, China) equipped with a 50 N load cell at room temperature; cylindrical hydrogel samples (diameter 10 mm × height 5 mm) were prepared using silicone molds and equilibrated in PBS (pH 7.4) for 24 h prior to testing.

### 2.6 Isolation and culture of TDSCs

Sprague-Dawley(SD) rats weighing 200 g and 6 weeks old were selected for the study. TDSCs were isolated and cultured from the rats following previously described methods [39]. Briefly, The rat Achilles tendon tissue was obtained and cut into tissue segments. The Tendon tissue samples were enzymatically digested using 3 mg/mL type I collagenase (C8140, Solarbio, Beijing, China) at 37 °C for 4 h in a temperature-controlled incubator. Following digestion, the cell suspension was sequentially processed through a 70 μm nylon mesh filter, subjected to centrifugation, and subsequently resuspended in complete culture medium. The cell suspension was inoculated into culture dishes and maintained under standard conditions for 3 days, with the initial medium replacement performed after cell adhesion. All cellular experiments were conducted using passage 3 (P3) cells.

### 2.7 *In vitro* biocompatibility of hydrogels

Hydrogel biocompatibility was evaluated using CCK-8 assays and live/dead staining. TDSCs (3 × 10^4^ cells/well) were cultured in lower chambers of 24-well Transwell plates, with hydrogels at varying concentrations (5, 10, 15, 20 mg/mL DAT) and Z-D formulations (0.1 %, 0.25 %, 0.5 %, 1 % w/v ZnO NPs) placed in upper chambers. CCK-8 measurements were performed at 1, 3, and 5 days post-culture. Viability staining using 2 μM Calcein-AM and 4 μM EthD-1 (37 °C, 30 min dark incubation) was conducted at days 1 and 3, followed by visualization using fluorescence microscopy. To further observe cell morphological changes under inflammatory conditions, an *in vitro* inflammation model was established, DAT hydrogels and Z-D hydrogels (200 μL/well) were pre-placed in 24-well plates. TDSCs (5 × 10^4^ cells/well) were seeded onto the hydrogel surfaces and cultured for 24 h in medium containing 1 μg/mL lipopolysaccharide (LPS; L8880, Solarbio, China) to simulate an inflammatory microenvironment. Following fixation with 4 % paraformaldehyde (PFA, 30 min at room temperature), cells were PBS-washed thrice and subsequently stained with TRITC-phalloidin (CA1610; Solarbio) followed by DAPI (C0065; Solarbio), with each 30-min incubation performed in the dark. Cytoskeletal morphology was analyzed using fluorescence microscopy.

### 2.8 Cell migration assays

Cellular migration capacity was assessed through scratch wound and Transwell migration assays. In the scratch assay, TDSCs (4 × 10^6^ cells/well) were cultured in 6-well Transwell chambers until reaching full confluency. Three parallel scratches were generated in monolayers using sterile 200 μL pipette tips, followed by three PBS (pH 7.4) washes to remove dislodged cells. followed by incubation with LPS-containing medium (1 μg/mL) to simulate inflammatory conditions. DAT hydrogels and Z-D hydrogels (500 μL each) were placed in the upper chambers. Wound closure was monitored at 0, 12, 24, and 48 h, with migration rates quantified using ImageJ software. The migration rate was calculated as: Migration rate (%) = (W_0_ - W_t_)/W_0_ × 100 %, where W_0_ represents the initial wound width at 0 h and W_t_ denotes the width at time t (t = 12, 24, 48 h).

Transwell migration assays were conducted by seeding TDSCs (4 × 10^6^ cells/well) in upper chambers of 6-well plates, with lower chambers containing 500 μL of DAT or Z-D hydrogels under inflammatory conditions. Following 12 h co-culture, non-migrated cells were mechanically removed using PBS-moistened swabs. Migrated cells underwent fixation with 4 % PFA (30 min), crystal violet staining (0.1 %, 20 min), and triple PBS rinses. Cell quantification was conducted by enumerating cells in three randomly chosen microscopic fields for each sample using an inverted phase-contrast microscope.

### 2.9 Verification of hydrogel anti-inflammatory properties

To validate the anti-inflammatory properties of hydrogels, immunofluorescence analysis was conducted using a Transwell co-culture system. TDSCs cultured in 24-well plate lower chambers were exposed to DAT or Z-D hydrogels in upper chambers, with inflammatory stimulation induced by 24-h LPS treatment (1 μg/mL). Upon medium removal, cellular fixation was performed using 4 % PFA at room temperature for 30 min, subsequently washed three times with PBS (5 min per wash). After blocking with immunostaining blocking buffer (Beyotime, China) for 1h at room temperature, cells were incubated overnight at 4 °C with primary antibodies:IL-6 antibody (Proteintech, China) and TNF-α antibody (Abcam, USA). The next day, cells were treated with 488-conjugated Immunofluorescent secondary antibody (Proteintech, China) for 2 h at 37 °C in the dark. Following three PBS washes, The samples were sequentially incubated with TRITC-phalloidin under light-protected conditions for 30 min, followed by nuclear counterstaining with DAPI solution (Solarbio, China) for 10 min. Fluorescence imaging was conducted using an inverted fluorescence microscope. For quantitative analysis of IL-6 and TNF-α expression levels, ImageJ software was employed, with three randomly selected fields quantified for each sample to ensure statistical reliability.

### 2.10 Medium induction of mouse mononuclear macrophage leukemia cells(RAW264.7)

The medium from TDSCs and TDSCs/Z-D hydrogel co-cultures was collected and centrifuged to prepare supernatants for inducing RAW264.7 cells; briefly, RAW264.7 cells were seeded in 24-well plates at a density of 5 × 10^4^ cells/well. In the control group, cells received no treatment. In the induction groups, cells were treated with supernatants from either TDSCs or TDSCs/Z-D hydrogel co-cultures (supernatant from TDSCs/Z-D hydrogel co-culture was mixed with complete RAW264.7 cell culture medium at a 1:1 ratio by volume), followed by qRT-PCR analysis to detect the expression of iNOS and TGF-β in RAW264.7 cells, with β-actin serving as the housekeeping gene; our study focused on 3 day.

### 2.11 Gene expression analysis

Total RNA extraction was carried out following the methodology detailed in reference [[32], [40]], with three biological replicates per group. The experimental procedures were performed, Total RNA was extracted using TRIzol reagent (P40927; Takara, China) according to the manufacturer's protocol. Subsequently, cDNA synthesis was performed through reverse transcription using a PrimeScript™ RT reagent kit (AT311; TRANSGEN BIOTECH, China) in a 20 μL reaction volume. Quantitative real-time PCR (qPCR) analysis was carried out using SYBR® Green Master Mix (P41014; TRANSGEN BIOTECH, Beijing) on a QuantStudio 5 Real-Time PCR System (Applied Biosystems, USA). Gene-specific primers (Table 1), designed using Primer 3.0 software, were employed for amplification. β-actin served as the endogenous control for normalization of gene expression data. The relative quantification of pro-inflammatory cytokine mRNA levels (IL-6, TNF-α, and IL-1β) was determined using the comparative threshold cycle (2−ΔΔCt) method, likewise,The relative quantification of Tendon tenogenic differentiation cytokine mRNA levels (SCX、TNMD and COL1) was determined using the comparative threshold cycle (2−ΔΔCt) method with triplicate measurements performed for each sample to ensure experimental reproducibility.

**Table 1:** Primer sequences.

Genes	Primer sequences (5′-3′)
IL-6	F:CCGTTTCTACCTGGAGTTTG
	R:GTTTGCCGAGTAGACCTCAT
TNF-α	F:CACCACGCTCTTCTGTCTACTG
	R:GGGCTACGGGCTTGTCACTC
IL-1β	F:CCAGGATGAGGACCCAAGCA
	R:TCCCGACCATTGCTGTTTCC
SCX	F:GACCCGCTTTCTTCCACAGC
	R:GTCACGGTCTTTGCTCAACTTT
TNMD	F:GTGATTTGGGTTCCCGCAGAA
	R:GTGGGATTGATCCAGTACATGG
COL1	F:CGAGTATGGAAGCGAAGG
	R:AGTGATAGGTGATGTTCTGG
iNOS	F:GTTCTCAGCCCAACAATACAAGA
	R:GTGGACGGGTCGATGTCAC
TGF-β	F:CCACCTGCAAGACCATCGAC
	R:CTGGCGAGCCTTAGTTTGGAC
β-actin	F:AGATGTGGATCAGCAAGCAG
	R:GCGCAAGTTAGGTTTTGTCA

### 2.12 Animal study

#### 2.12.1 Minimally invasive surgical procedures

All animal experiments were carried out in strict adherence to the ethical guidelines established by the Animal Ethics Committee of Ningbo University(Approval No. 13876). Sixty male Sprague-Dawley rats, obtained from the Experimental Animal Center of Ningbo University, China, were utilized in this study. Twelve 6-week-old rats were allocated for TDSCs isolation, while 48 age-matched rats (body weight 200 ± 20 g) were randomly assigned to four experimental groups (n = 12 per group) for *in vivo* tendon repair assessment (1): Normal group (2): AT model group: received percutaneous injection of 30 μL type I collagenase solution (concentration 10 mg/mL; Solarbio, China) into the mid-portion of Achilles tendons to induce tendinopathy (3): DAT group: administered intralesional injection of 200 μL DAT hydrogel (15 mg/mL) at 1 week post-modeling (4): Z-D group: treated with 200 μL 0.25 % Z-D hydrogel injection at 1 week post-modeling. For histological and molecular analyses, animals were humanely euthanized at 1 and 4 weeks post-operation (n = 6 per time point).

#### 2.12.2 Gait analysis

To comprehensively evaluate the therapeutic potential of hydrogels for Achilles tendon treatment, the Achilles functional index (AFI) was employed to quantify tendon mobility. The experimental protocol was conducted at 1 and 4 weeks post-surgery, the hind paw pads of rats were coated with non-toxic ink (Solarbio, China) and allowed to walk freely in a standardized corridor (15 cm width × 100 cm length) to obtain continuous footprint patterns. Key parameters were measured using a digital caliper (Mitutoyo, Japan).Morphometric measurements were performed as follows: Print Length (PL) was defined as the vertical distance from the tip of the third toe to the most posterior point of the heel. Toe Spread (TS) represented the maximal horizontal distance between the medial aspect of the first toe and the lateral aspect of the fifth toe. Intermediate Toe Spread (IT) was measured as the horizontal distance between the second and fourth toes. Functional indices were calculated using the following standardized formulas (1): Print Length Factor (PLF) = (NPL - EPL)/EPL (2):Intermediate Toe Spread Factor (ITF) = (EIT - NIT)/NIT (3): Toe Spread Factor (TSF) = (ETS - NTS)/NTS (4): Achilles functional index (AFI) = 74(PLF) + 161(TSF) + 48(ITF), where N denotes the mean value obtained from the healthy control group and E represents the experimental measurements. Morphometric data were collected from five rats per group and statistically analyzed, with the results were statistically evaluated and expressed as mean ± SEM.

#### 2.12.3 Real-time superb microvascular imaging (SMI) ultrasonography

This study employed real-time SMI ultrasonography to evaluate local blood supply in Achilles tendons, with identical imaging parameters (including scanning depth, focal position, and time gain compensation) as conventional B-mode ultrasonography. For quantitative assessment of tendon tissue, the vascular index (VI) surrounding each Achilles tendon was obtained through SMI technology. The operational protocol consisted of the following steps: During SMI, the operator manually delineated a region of interest (ROI) along the lesion margin encompassing maximal Doppler signals. The VI value was subsequently automatically calculated using dedicated analytical software installed on US-manufactured equipment. This quantitative index was calculated as the percentage ratio of Doppler signal-positive pixels to the total number of pixels within the defined ROI. To ensure measurement reliability, three independent measurements were performed by the same observer on identical Achilles tendons, with intra-observer consistency statistically evaluated [41].

#### 2.12.4 Biomechanical testing

Achilles tendon specimens were harvested from each experimental group(n = 3 per group) at 1 and 4 weeks postoperatively and subjected to biomechanical evaluation using an electromechanical testing system(Model #3366, Instron Corporation, Shanghai, China). Use an integrated pneumatic grip to secure the Achilles tendon, with the system equipped with a precision load cell (0–200 N range). Initial measurements included documentation of Achilles tendon length and cross-sectional dimensions. The cross-sectional area (S) was calculated by elliptic approximation formula:S = π × M1 × M2/4, where M1 represents maximum tendon width (mm) and M2 denotes maximum tendon thickness (mm). Uniaxial tensile testing was performed in displacement-control mode at 10 mm/min. Biomechanical parameters were extracted from load-displacement curves (1):Maximum load(N): Peak failure force (2):Stiffness(ST):ST = N/M3 (3):Young's modulus(YM):YM = σ/ε; where strain(ε) was calculated as: ε = (D/OL) × 100 %; Parameter definitions:M3 = displacement at maximal load (mm); σ = stress(MPa); calculated as load divided by original cross-sectional area; D = absolute deformation (mm); OL = initial gauge length (mm).

#### 2.12.5 Histological analysis

At designated time points (1 and 4 weeks post-surgery), Sprague-Dawley rats were humanely euthanized, and Achilles tendon tissues were collected. The specimens were immediately immersed in 4 % PFA and fixed at 4 °C for 48 h. Subsequently, the samples underwent sequential dehydration in graded ethanol solutions (70 %, 80 %, 90 %, and 100 %), followed by xylene clearing and paraffin embedding. Using a rotary microtome, consecutive tissue sections of 5 μm thickness were obtained for further analysis. Multiple staining protocols were employed for comprehensive histological evaluation, Hematoxylin and eosin (HE) staining for assessment of cellular morphology and tissue architecture; Masson's trichrome staining for visualization of collagen fiber organization and extracellular matrix composition. Immunohistochemical and immunofluorescence staining were subsequently performed to investigate specific protein expression patterns. All stained sections were mounted with neutral resin and examined under a light microscope equipped with digital imaging capabilities, with particular attention to cellular infiltration, collagen alignment, and vascularization patterns.The immunohistochemical workflow involved sequential steps of slide deparaffinization, antigen retrieval via citrate buffer (Solarbio, China) microwave treatment, endogenous peroxidase blockade with 3 % H_2_O_2_ (10 min), 37 °C trypsin digestion (2 h), and 10 % goat serum blocking, Followed this step, the sections were subjected to overnight incubation at 4 °C with primary antibodies targeting SCX (PA5-115874, Invitrogen), TNMD (ab203676, Abcam), COL I (A16891, Proteintech), COL III (A3795, Proteintech), IL-6 (21865-1-AP, Proteintech), TNF-α (21865-1-AP, Proteintech), CD86(13395-1-AP,Proteintech)and CD206(18704-1-AP,Proteintech). Detection employed HRP-conjugated secondary antibody (CR2107161, Servicebio) with DAB chromogenic development (CR2103183,Servicebio).For immunofluorescence, 488-conjugated(or 594-conjugated) secondary antibody was applied under light-protected conditions for 1 h, followed by DAPI nuclear counterstaining (C0065, Solarbio, 5 min), with fluorescence-preserved specimens imaged via confocal laser microscopy. Histopathological evaluation utilized standardized scoring criteria with normal specimens designated as baseline (score = 0), while tendon adhesion severity was quantitatively assessed through triple-blind independent evaluations.

#### 2.12.6 *In vivo* biocompatibility

At designated time points of 1 and 4 weeks post-operation, experimental rats were humanely euthanized through intraperitoneal administration of anesthetic overdose. A complete necropsy was performed, with systematic collection of major organs (heart, liver, spleen, lungs, and kidneys) for macroscopic examination and high-resolution photographic documentation. The harvested organs were immediately fixed in 4 % PFA solution at 4 °C for 48 h, followed by standard paraffin embedding and sectioning at 5 μm thickness.This systematic histopathological assessment was conducted to evaluate potential systemic toxicity and biocompatibility of the implanted biomaterial.

#### 2.12.7 Statistical analysis

Experimental data obtained from rats that did not survive the complete study duration were systematically excluded from subsequent analyses. Statistical comparisons were performed using appropriate parametric tests: Statistical analyses were performed using one-way analysis of variance (ANOVA) with Tukey's post hoc test for multiple group comparisons or Student's t-test for comparisons between two groups. All statistical computations were executed using GraphPad Prism software (version 9.0). A p-value <0.05 was considered statistically significant, with significance levels denoted as follows: ∗*p* < 0.05, ∗∗*p* < 0.01, and ∗∗∗*p* < 0.001. Experimental data are expressed as mean ± standard error of the mean (SEM), with the number of replicates (n) specified for each experimental condition.

## 3 Results

### 3.1 Preparation and characterization of hydrogels

**Fig. 1:**
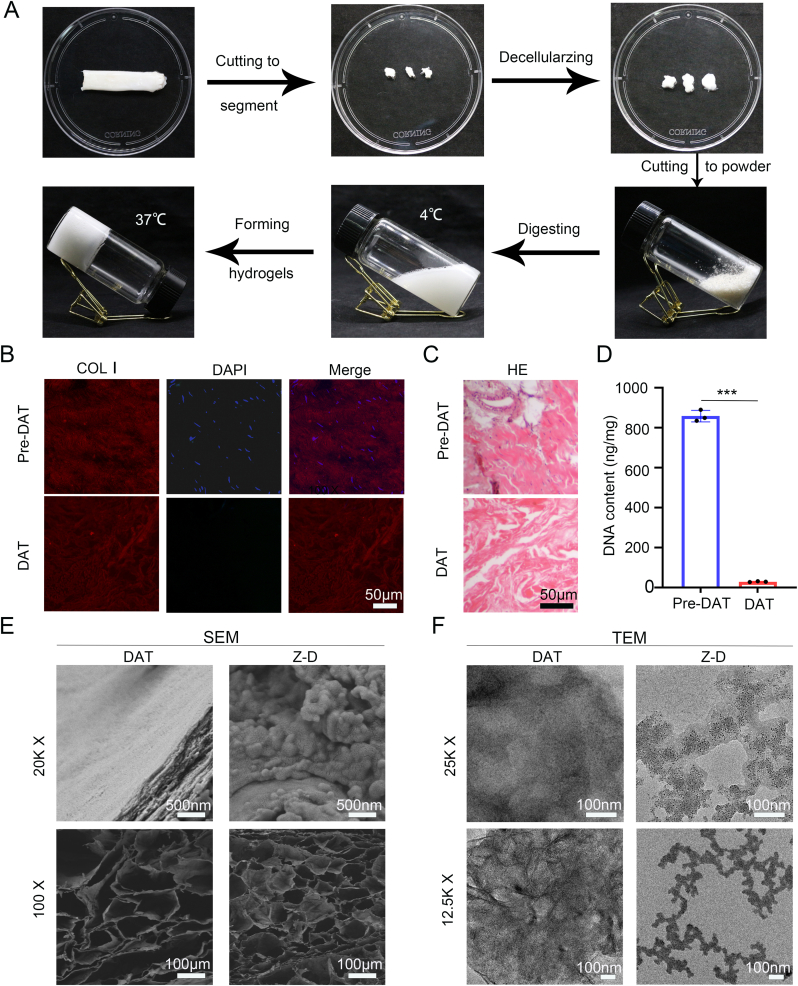
Characterization of DAT hydrogels. (A) Schematic diagram of DAT hydrogel preparation. (B) Immunofluorescence staining images showing COL I distribution and nuclei in native Achilles tendon (pre-DAT) and DAT hydrogel. (C) Comparative hematoxylin-eosin (HE) staining of tissues pre-DAT and DAT hydrogel. (D) Quantitative analysis of residual DNA content pre-DAT and DAT hydrogel. (E) SEM images of DAT hydrogel and Z-D hydrogel at 100 × and 20000× magnifications. (F) TEM of DAT hydrogel and Z-D hydrogel at 25000 × and 12500× magnifications. (∗*p* < 0.05, ∗∗*p* < 0.01, and ∗∗∗*p* < 0.001).

Based on the optimized decellularization protocol, we successfully prepared a 15 mg/mL DAT hydrogel system. This system maintained liquid characteristics at 4 °C and spontaneously formed a gel state at 37 °C (physiological temperature). Systematic evaluation demonstrated that the DAT hydrogel exhibited excellent thermoresponsive injectability and shape adaptability to meet the requirements for injectable hydrogels in biomedical applications, enabling precise filling of irregular tissue defects (Fig. 1A). Immunofluorescence and HE staining results after decellularization confirmed the intact preservation of type I collagen fiber structures while showing significantly reduced DNA content (Fig. 1B–D), verifying that the process effectively removed cellular components while maintaining the integrity of the extracellular matrix.

Through physical integration of ZnO NPs into the DAT hydrogel, we successfully constructed a Z-D hydrogel system. Scanning electron microscopy (SEM) characterization revealed that both pure DAT hydrogel and Z-D hydrogel after lyophilization exhibited typical three-dimensional porous network structures at 100 × and 20000 × magnifications (Fig. 1E). Importantly, the integration of ZnO NPs maintained the intrinsic microstructural architecture of the matrix, as evidenced by scanning electron microscopy (SEM) analysis demonstrating homogeneous nanoparticle dispersion throughout the matrix.,Further verification in DAT hydrogels with different concentrations and 15 mg/mL DAT loaded with ZnO NPs demonstrated stable pore structures and nanoparticle distribution (Fig. S1A). Transmission electron microscopy (TEM) further revealed that ZnO NPs were uniformly dispersed as spherical particles with diameters <100 nm in the hydrogel matrix (Fig. 1F). The measured lattice spacing matched standard ZnO crystallographic parameters, confirming the structural integrity of the nanoparticles.

**Fig. 2:**
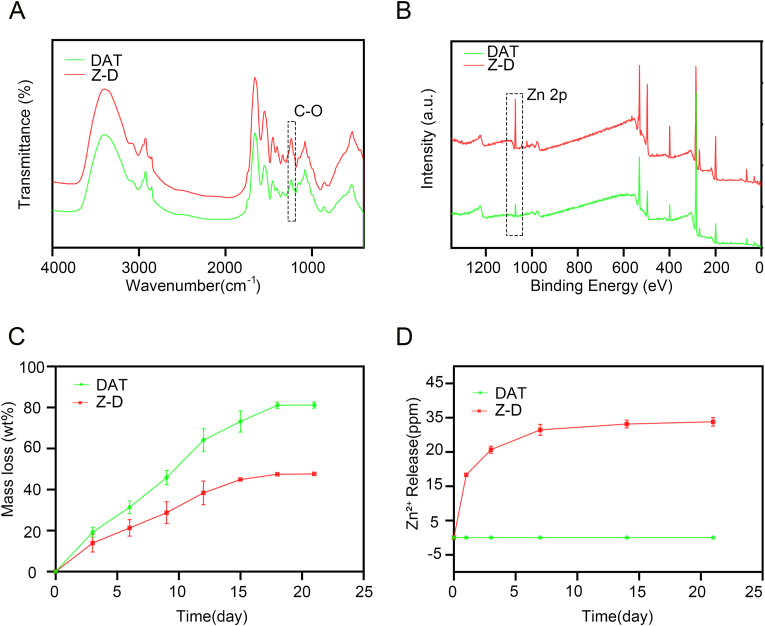
Characterization of Z-D hydrogels. (A) Comparative FTIR of DAT hydrogel and Z-D hydrogel. (B) XPS survey scans of DAT hydrogel and Z-D hydrogel. (C) *In vitro* degradation profiles of DAT hydrogel and Z-D hydrogel. (D) *In vitro* release profiles of DAT hydrogel and Z-D hydrogel (∗*p* < 0.05, ∗∗*p* < 0.01, and ∗∗∗*p* < 0.001).

Fourier-transform infrared (FTIR) spectroscopy analysis (Fig. 2A) showed a significant enhancement of the C-O bond vibration peak at 1238 cm^−1^ in Z-D composite hydrogels, indicating coordination interactions between Zn^2+^ and phenolic hydroxyl groups in the DAT matrix. XPS analysis (Fig. 2B) demonstrated characteristic Zn 2p peaks at a binding energy of 1021 eV in the composite system, in addition to elemental composition of C, N, and O from the DAT matrix, further confirming the successful loading of ZnO NPs.The compressive mechanical properties of the hydrogels (Fig. S1B and C) reveal that the Z-D hydrogel exhibits a compressive modulus of 21.32 kPa, significantly higher than the 12.79 kPa of the pure DAT hydrogel; this 160 % modulus enhancement originates from ionic crosslinking between ZnO NPs and carboxyl groups of the DAT matrix.

*In vitro* degradation kinetics studies (Fig. 2C) revealed that in PBS containing 0.02 mg/mL type I collagenase, the control DAT hydrogel showed 71 % mass loss after 15 days, while the Z-D hydrogel exhibited a significant reduction to 44 %. This difference was attributed to enhanced network crosslinking density through metal-ligand interactions between Zn^2+^ and polyphenol groups. *In vitro* ion release studies (Fig. 2D) demonstrate that the Zn^2+^ release profile from the Z-D hydrogel in PBS exhibits a typical two-phase pattern, an initial rapid burst release phase (within 1 day) followed by a sustained slow-release phase (over 7 days): the burst release phase achieved a concentration of 18.3 ppm within 1 day, while the sustained release phase showed a significantly reduced release rate, reaching a cumulative concentration of 31.2 ppm by 7 day. This release behavior is likely regulated synergistically by the large pore structure of the hydrogel and the electrostatic retention within the DAT matrix, ensuring the synergistic integration of early rapid anti-inflammatory response with mid-to-late-stage regenerative support functions. These results demonstrate that the Z-D hydrogel not only achieves stable loading of functional nanomaterials but also obtains controllable degradation properties through crosslinking regulation, providing theoretical support for its long-term applications in tissue repair and related fields.

### 3.2 *In vitro* cell viability, proliferation, and migration

**Fig. 3:**
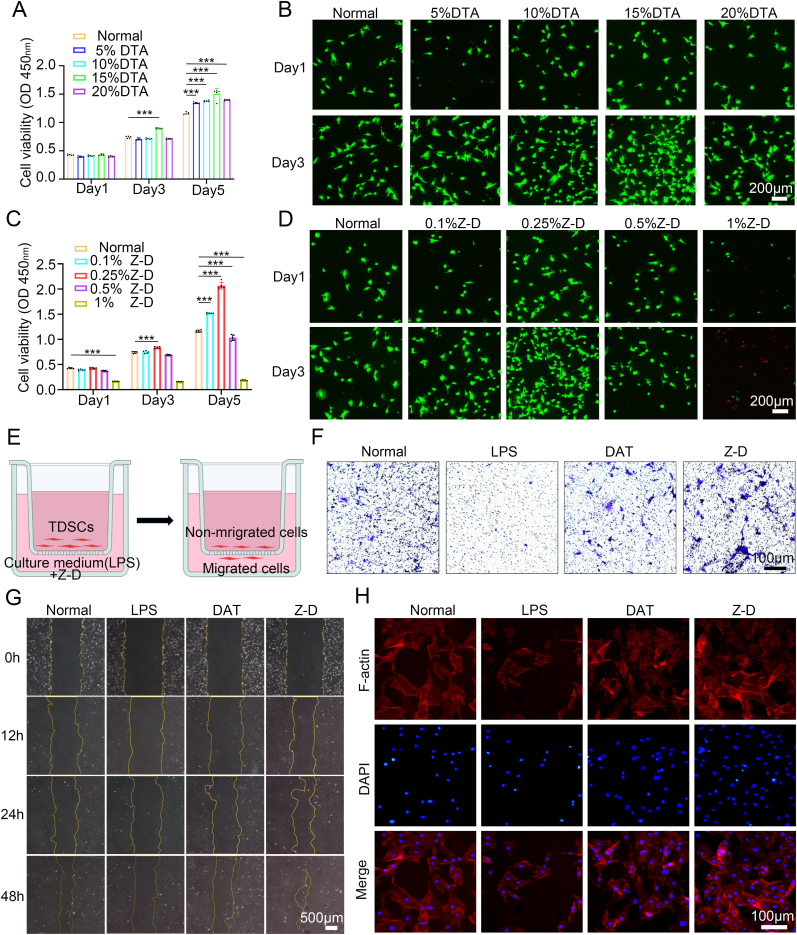
*In vitro* cytocompatibility and cell migration properties of hydrogels. (A) Effects of DAT hydrogels at different concentrations on TDSCs viability; (B) Live/dead cell staining images of TDSCs treated with DAT hydrogels at different concentrations; (C) Effects of Z-D hydrogels at different concentrations on TDSCs viability; (D) Live/dead cell staining images of TDSCs treated with Z-D hydrogels at different concentrations; (E) Schematic diagram of Transwell assay for detecting vertical cell migration; (F) Vertical migration images from Transwell assays in different groups; (G) Horizontal migration images from scratch assays; (H) Cytoskeleton staining showing cell adhesion and spreading status. (∗*p* < 0.05, ∗∗*p* < 0.01, and ∗∗∗*p* < 0.001).

We successfully isolated TDSCs and performed immunofluorescence identification for CD29,CD44, and CD90 (Fig. S2).Cytocompatibility of hydrogels was evaluated using CCK-8 assays and Live/Dead staining with rat TDSCs. After culturing TDSCs on DAT hydrogel surfaces for 1, 3, and 5 days, CCK-8 results demonstrated that all DAT hydrogel concentrations (5 %, 10 %, 15 %, 20 % w/v) promoted cell growth, with 15 mg/mL DAT showing optimal performance (Fig. 3A). To simulate *in vivo* hydrogel release dynamics, TDSCs were cultured in Transwell lower chambers with hydrogels placed in upper chambers. Live/Dead staining after 1 and 3 day co-culture confirmed that hydrogel concentrations did not impair proliferation and viability, with 15 mg/mL DAT maintaining superior performance (Fig. 3B), therefore selected for subsequent cellular and animal experiments.

To identify the optimal ZnO NPs concentration, hydrogels incorporating 0.1 %, 0.25 %, 0.5 %, and 1 % (w/v) ZnO NPs were evaluated. Cell viability exhibited a concentration-dependent biphasic response, peaking at 0.25 % Z-D hydrogel (Fig. 3C). Notably, 1 % Z-D hydrogel significantly suppressed proliferation. Live/Dead staining further validated the superior cytocompatibility and pro-proliferative effects of 0.25 % Z-D hydrogel (Fig. 3D), establishing this formulation for further investigation.

TDSCs migration was assessed via Transwell assays and scratch wound healing experiments under inflammatory conditions induced by 1 μg/mL LPS treatment for 24 h (LPS group remained untreated). Transwell results (Fig. 3E) demonstrated vertical migration capacity: LPS-treated cells showed reduced transmigration rates compared to normal controls, while both DAT and Z-D hydrogel co-culture groups exhibited enhanced migration versus LPS group, with Z-D hydrogel showing maximal efficacy (Fig. 3F). Statistical analysis confirmed significant differences in vertical migration (Fig. S3A). Scratch assays (Fig. 3G) revealed horizontal migration, where Z-D hydrogel group achieved 90 % wound closure at 48 h versus 35 % in LPS group (Fig. S3B). Normal and DAT hydrogel groups showed comparable migration rates, indicating synergistic migration-promoting effects of DAT hydrogel and ZnO NPs under inflammation.

Cytoskeletal staining (Fig. 3H) showed restricted cell adhesion and spreading in LPS group, while DAT and Z-D hydrogel groups maintained normal adhesion morphology. Remarkably, Z-D hydrogel significantly outperformed LPS group in promoting cell adhesion and spreading, further confirming its biocompatibility advantages.

### 3.3 The anti-inflammatory and pro-differentiation functions of hydrogels *in vitro*

**Fig. 4:**
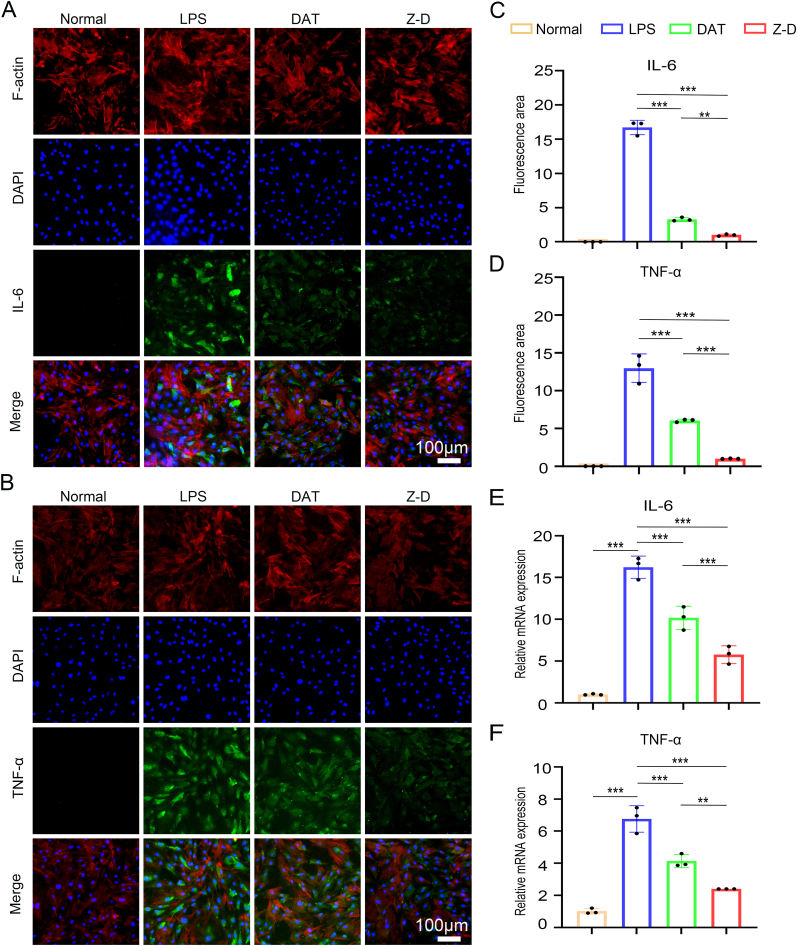
Inflammatory regulation of hydrogels. (A) IL-6 immunofluorescence staining in different groups under inflammatory induction; (B) TNF-α immunofluorescence staining in different groups under inflammatory induction; (C, D) Quantitative analysis of IL-6 and TNF-α immunofluorescence staining; (E, F) RT-qPCR results showing gene expression of IL-6 and TNF-α in the inflammatory microenvironment. (∗*p* < 0.05, ∗∗*p* < 0.01, and ∗∗∗*p* < 0.001).

For investigation of hydrogel-mediated anti-inflammatory responses in TDSCs under pro-inflammatory conditions, a Transwell co-culture platform was established, featuring TDSCs in the lower chamber and hydrogel materials in the upper chamber. An inflammatory microenvironment was induced by supplementing the culture medium with 1 μg/mL LPS, followed by 24-h incubation. Immunofluorescence analysis demonstrated distinct expression patterns: the LPS-treated group exhibited maximal expression of the pro-inflammatory cytokine IL-6, while the Z-D hydrogel group showed significantly reduced IL-6 fluorescence intensity, approaching baseline levels observed in the normal control group (Fig. 4A–C). The expression trend of another pro-inflammatory factor, TNF-α, paralleled that of IL-6, with the LPS group exhibiting the highest expression and the Z-D hydrogel group showing the lowest (Fig. 4B–D). Furthermore, RT-qPCR analysis demonstrated that the Z-D hydrogel group down regulated the gene expression levels of IL-6, and TNF-α compared to the LPS group (Fig. 4E and F). the similar downregulation of IL-1β was also found (Fig. S4A).To evaluate the promoting effect of hydrogels on TDSC differentiation, TDSCs were cultured in the lower chamber of Transwell plates with the hydrogel placed in the upper chamber for 3 consecutive days. RT-qPCR analysis further revealed that compared to the normal group, the Z-D hydrogel group exhibited significantly upregulated gene expression of SCX, TNMD, and COL1 (Fig. S4B–D), demonstrating superior outcomes to the DAT group.

### 3.4 Effect of TDSCs and TDSCs/Z-D hydrogel on macrophage polarization towards the M2 phenotype

Considering the ability of TDSCs and TDSCs/Z-D hydrogel to modulate macrophage polarization, the expression of M1 and M2 markers was assessed *in vitro*. RAW264.7 cells were stimulated with conditioned medium from TDSCs and TDSCs/Z-D hydrogel co-cultures; results showed that on 3 day post-stimulation, the relative expression level of TGF-β in the TDSCs/Z-D hydrogel group was significantly higher than in the TDSCs group, while conversely, the expression level of the pro-inflammatory gene iNOS in the TDSCs/Z-D hydrogel group was lower than in the TDSCs group (Fig. S4E and F).

### 3.5 *In Vivo* Evaluation using rat Achilles tendinopathy model

**Fig. 5:**
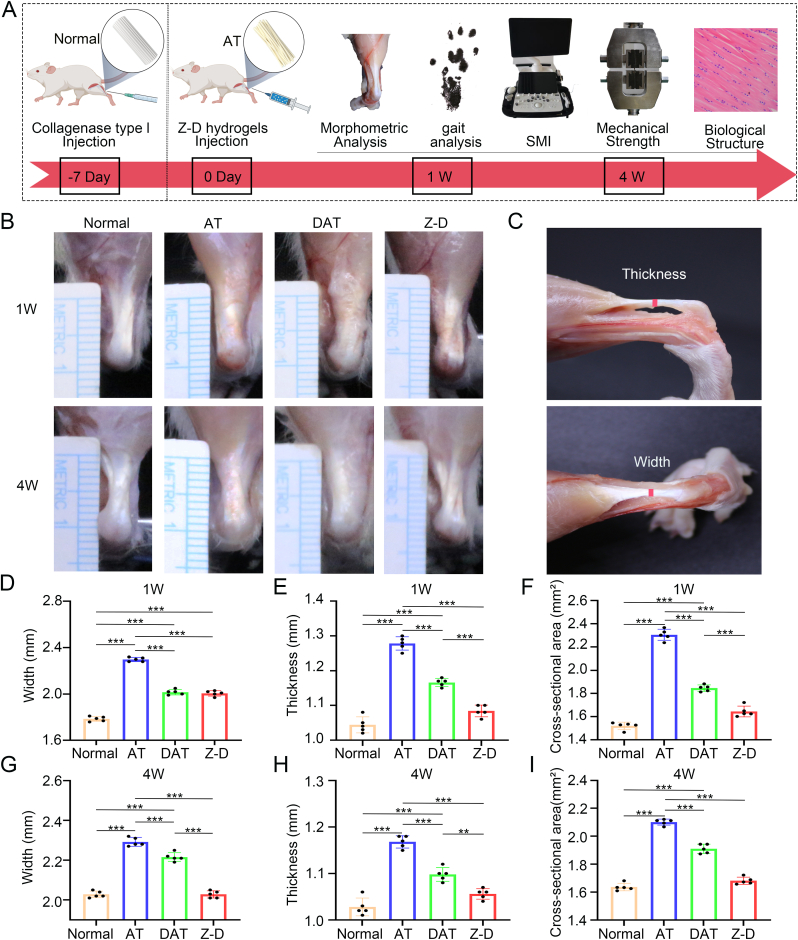
*In Vivo* Evaluation of Hydrogel-Mediated Achilles Tendinopathy Repair.(A) Schematic diagram of experimental workflow.(B) Macroscopic anatomical views of rat Achilles tendons. (C) Schematic representation of tendon thickness and width measurements. (D–I) Quantitative assessment of tendon width, thickness, and cross-sectional area was performed at 1 and 4 weeks post-operation. (∗*p* < 0.05, ∗∗*p* < 0.01, and ∗∗∗*p* < 0.001).

A rat Achilles tendinopathy model was surgically established (Fig. S5) [42], with DAT and Z-D hydrogels injected into the injured tendons at post-operative day 7. Experimental groups included: normal control, tendinopathy control (AT group, untreated), DAT-treated group, and Z-D-treated group. Comprehensive therapeutic effects were evaluated through morphological assessment, gait analysis, SMI ultrasonography, biomechanical testing, and histopathological analysis at 1 and 4 weeks post-operation (Fig. 5A).

#### 3.5.1 Morphological evaluation, gait analysis, SMI ultrasonography, and biomechanical testing

Macroscopic evaluation of tendon specimens (Fig. 5B) revealed structural differences across groups: Normal tendons maintained well-defined boundaries. The AT group exhibited severe damage at 1 week post-operation, characterized by opaque yellowish fibrotic tissue encapsulation and adhesion between disordered tendon bundles. DAT and Z-D treated groups demonstrated progressive repair features with reduced abnormal tissue deposition and visible tendon bundles. By 4 weeks, DAT and Z-D groups showed gradual absorption of pathological tissue, while the AT group developed irreversible tendon bundle fusion. Notably, the Z-D group achieved near-normal tendon morphology at 4 weeks.Through measurements of tendon width, thickness and cross-sectional area (Fig. 5C) at 1 week post-operation, the AT group exhibited significantly higher tendon width, thickness and cross-sectional area compared to the normal group, while the DAT and Z-D groups showed significantly lower parameters than the AT group (Fig. 5D–F). At the 4-week post-operative time point, the Z-D group demonstrated morphological parameters comparable to the normal control group and showed statistically significant improvements over the DAT group (Fig. 5G–I).

**Fig. 6:**
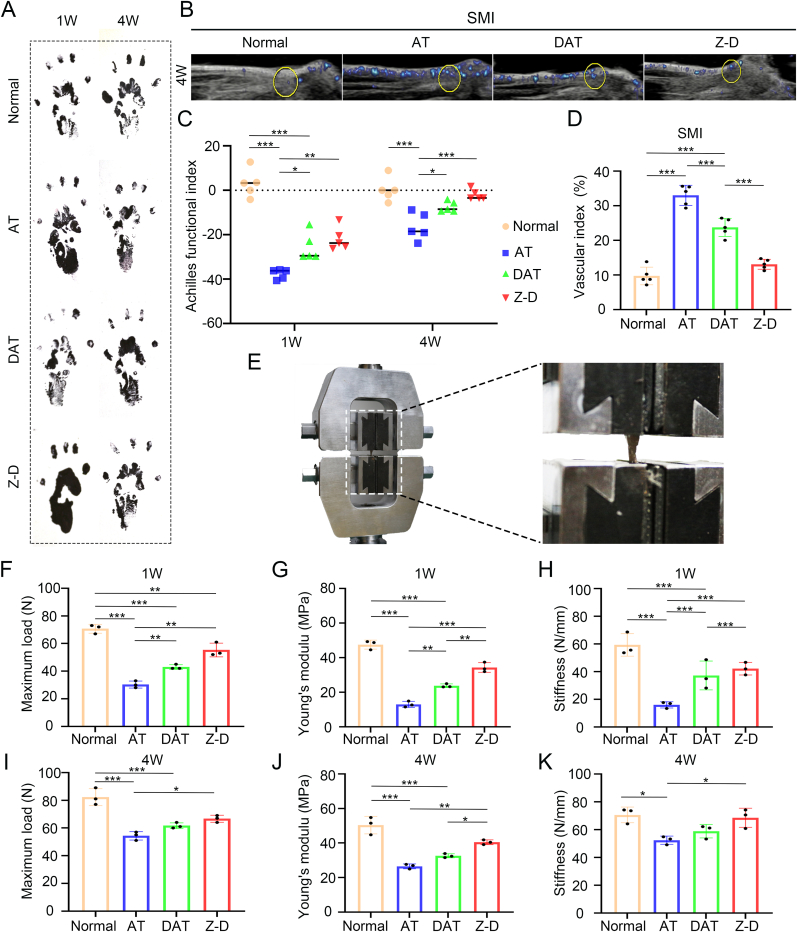
*In vivo* therapeutic evaluation of hydrogel in rat Achilles tendinopathy. (A) Representative paw prints; (B) Ultrasonic imaging of blood flow in the Achilles tendon region; (C) Quantitative analysis of AFI; (D)Quantitative analysis of Achilles tendon blood flow; (E) Achilles tendon using electromechanical testing system (F–K) Quantitative analysis of maximum load, Young's modulus, and stiffness in different groups at 1 and 4 weeks post-surgery. (∗*p* < 0.05, ∗∗*p* < 0.01, and ∗∗∗*p* < 0.001).

We further evaluated the restored motor function, real-time SMI ultrasound, and mechanical properties following hydrogel treatment. Gait analysis (Fig. 6A) revealed significantly prolonged paw print lengths in the AT group at 1 week post-operation, indicative of movement dysfunction from tendinopathy. Both DAT and Z-D groups exhibited progressive paw print shortening by 4 week. Quantification using the Achilles functional index (AFI) demonstrated significantly lower AFI values in the AT group versus normal controls at both 1 and 4 week timepoints (Fig. 6C). While DAT and Z-D groups showed moderate AFI recovery at 1 week, the Z-D group achieved near-normal AFI levels by 4 week.

Real-time SMI (Fig. 6B) detected increased blood perfusion in all injured groups at 4 weeks, likely reflecting compensatory angiogenesis in inflammatory microenvironments. However, DAT and Z-D groups showed significantly lower perfusion than the AT group, with the Z-D group achieving near-normal perfusion levels (Fig. 6D).

Biomechanical testing using a electromechanical testing system (Fig. 6E) demonstrated severe reductions in maximum load, Young's modulus, and stiffness for the AT group at 1 week (Fig. 6F–K). Although DAT and Z-D groups initially showed mechanical property declines, the Z-D group exhibited superior recovery trajectories. By 4 weeks, Z-D tendons displayed maximal restoration of mechanical properties, approaching normal control values and significantly outperforming the DAT group.Finally, histological repair score and quantitative analysis of adhesion degree (Fig. S6A and B) were also performed in all groups, which also verified the maximum repair effect of Z-D group.

#### 3.5.2 Histological analysis

**Fig. 7:**
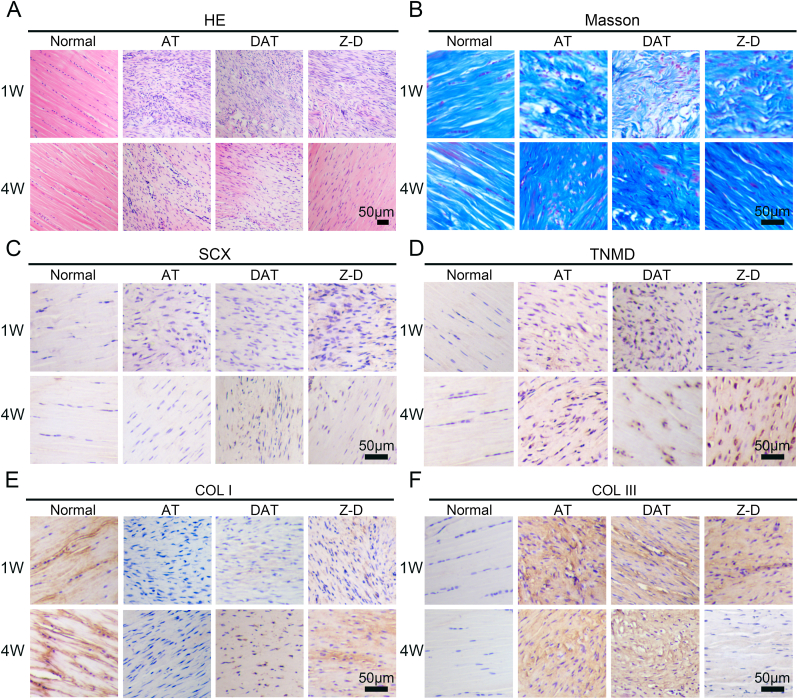
Histological analysis. (A) HE staining; (B) Masson staining; (C) Immunohistochemistry of SCX in each group at early repair stage (1 week) and late stage (4 weeks); (D) Immunohistochemistry of TNMD expression changes. (E) Immunohistochemistry of COL I during injury repair; (F) Immunohistochemistry of COL III during injury repair.

**Fig. 8:**
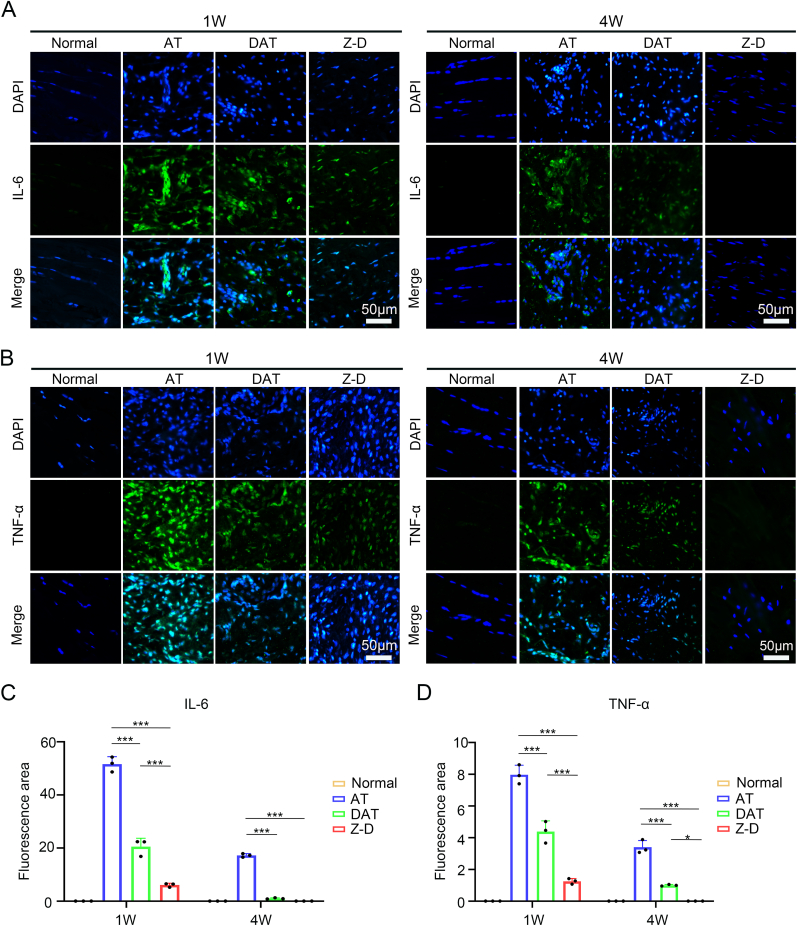
Immunofluorescence Analysis.(A) IL-6 immunofluorescence staining in Achilles tendon tissues; (B) TNF-α immunofluorescence staining in Achilles tendon tissues; (C–D) Quantitative immunofluorescence analysis of IL-6 and TNF-α inflammatory expression intensities. (∗*p* < 0.05, ∗∗*p* < 0.01, and ∗∗∗*p* < 0.001).

Histological analyses including HE staining, Masson's trichrome staining, and immunohistochemistry were performed to elucidate tissue microstructure and healing mechanisms. HE staining (Fig. 7A) revealed severely disordered collagen fiber alignment in the AT group at 1 week post-operation. Both DAT and Z-D groups exhibited improved repair outcomes, with Z-D group demonstrating superior collagen morphology and alignment regularity compared to DAT group, though remaining distinct from normal controls. By 4 weeks, collagen organization progressively normalized in DAT and Z-D groups, with Z-D group achieving near-normal alignment patterns. Masson's trichrome staining (Fig. 7B) showed significantly compromised collagen integrity in the AT group at 1 week, while DAT and Z-D groups displayed partial restoration. At 4 weeks, the AT group maintained limited collagen repair, DAT group achieved moderate recovery, and Z-D group exhibited collagen architecture closest to normal tendons.Immunohistochemical analysis of tendon differentiation markers revealed distinct expression patterns. The Z-D group exhibited significantly elevated SCX expression at 1 week relative to the AT group. (Fig. 8C), indicating enhanced tenogenic progenitor differentiation. All groups showed declining SCX expression by 4 weeks, consistent with its role in early-stage tendon repair. Conversely, TNMD expression remained at baseline levels across groups at 1 week, though Z-D group maintained significantly higher expression than AT group. By 4 weeks, TNMD expression markedly increased in DAT and Z-D groups, peaking in Z-D group (Fig. 8D), confirming sustained activation of tendon maturation markers.Collagen remodeling analysis showed differential COL I expression at 1 week: AT group exhibited lowest expression, while Z-D group demonstrated significantly higher levels than other experimental groups (Fig. 8E). Z-D group further increased COL I deposition by 4 weeks, achieving histological features closest to normal tendons. COL III displayed inverse dynamics, with peak expression in AT group at 1 week and minimal expression in Z-D group. All groups showed significant COL III downregulation by 4 weeks, most pronounced in Z-D group (Fig. 8F), aligning with physiological collagen transition during tendon healing.

To investigate the mechanism of Z-D hydrogels in Achilles tendon repair, we assessed their anti-inflammatory effects using immunofluorescence staining for IL-6 and TNF-α. The findings revealed (Fig. 8A) the AT group exhibited significantly higher IL-6 expression intensity compared to DAT and Z-D groups at 1 week post-operation, with the Z-D group showing the lowest expression. By 4 weeks post-operation, IL-6 levels decreased across all groups, the Z-D group maintained minimal expression. TNF-α expression followed a similar pattern to IL-6 (Fig. 8B), with the Z-D group demonstrating the lowest expression at 1 week and approaching normal levels by 4 weeks. Combined with quantitative analysis of the aforementioned results (Fig. 8C and D), these findings suggest that Z-D hydrogels synergistically suppress inflammatory factor expression through sustained Zn^2+^ release, thereby accelerating tissue regeneration. In the study of immune cell mechanisms (Fig. S7A and B), we performed immunofluorescence staining for the macrophage M1 marker CD86 and the M2 marker CD206 to further illustrate that early Zn^2+^ release (day 7 post-operation) suppresses M1 macrophage expression; the Z-D hydrogel group exhibited the lowest CD86 expression and the highest CD206 expression, demonstrating that the early Z-D hydrogel promotes polarization of macrophages from the M1 to M2 phenotype, at 4 weeks, CD206 was not expressed in the Z-D hydrogel group, just like in the normal group, while there was still expression in the AT group. thereby shifting the inflammatory microenvironment toward a pro-repair state.The results indicate that Z-D hydrogels establish stable intracellular zinc homeostasis *in vivo* via a controlled Zn^2+^ release system. This dynamic ionic regulation mechanism temporally activates repair-related pathways during the early phase, it induces SCX upregulation and moderately promotes COL III synthesis, while in the late phase, it significantly enhances TNMD expression and accelerates COL I deposition. Notably, the Z-D group demonstrated superior tendon repair capability compared to other experimental groups, confirming zinc's pivotal role in tendon stem cell recruitment, lineage-specific differentiation induction, and anti-inflammatory microenvironment establishment, thereby providing an innovative therapeutic strategy for functional regeneration in tendinopathy.

#### 3.5.3 *In vivo* biosafety evaluation

Gross examination and HE staining of major organs (heart, liver, spleen, lung, kidney) revealed no morphological abnormalities and pathological lesions in DAT and Z-D groups (Fig. S8A and B). All experimental groups maintained well-preserved histological architecture with intact parenchymal cell morphology, showing no significant differences compared to normal controls, which confirms the excellent biocompatibility of the hydrogel systems.

## 4 Discussion

The creation of innovative minimally invasive therapies for AT addresses a crucial unmet clinical demand [43,44] Targeting both tendon regeneration and anti-inflammatory effects as dual therapeutic goals for AT holds significant importance compared to traditional systemic pharmacological therapies, minimally invasive local drug delivery not only enhances targeting efficiency and reduces systemic side effects [45,46], but also prevents secondary tissue injury and surgical pain linked to open procedures. However, existing therapeutic technologies have yet to establish a complete minimally invasive treatment system for AT. Advances in tissue engineering and regenerative medicine have provided innovative strategies for AT repair and regeneration [[47], [48], [49]]. Against this background, this study innovatively developed a ZnO NPs-loaded Z-D hydrogel system to achieve synergistic therapy through modulation of the inflammatory microenvironment and promotion of tendon regeneration.

The design of the Z-D hydrogel integrates the temperature-sensitive gelation properties of DAT hydrogel with the functional advantages of ZnO NPs. Experimental results confirmed that the excellent biocompatibility of DAT hydrogel and its ability to support TDSCs proliferation and differentiation were preserved [[50], [51]], while the sustained-release characteristics of ZnO NPs ensured continuous Zn^2+^ release.Concurrently, the large-pore structure of the hydrogel provides efficient diffusion pathways for ZnO NPs, while the natural components of dECM achieve sustained Zn^2+^ release through electrostatic interactions, synergistically ensuring the material's biological safety and degradability to meet its functional needs as a drug delivery carrier. This dual-functional design not only provides an optimal microenvironment for TDSCs but also directly exerts Zn^2+^- mediated anti-inflammatory and pro-regenerative effects at the injury site, aligning with the core requirements of minimally invasive therapy [[52], [53]].

Notably, the inhibitory effect of chronic inflammatory microenvironments on TDSCs activity constitutes a major obstacle to tendon repair [54]. Using an LPS-induced *in vitro* inflammatory model, this investigation revealed that the Z-D hydrogel markedly suppressed the expression of key pro-inflammatory mediators, including IL-6, TNF-α, and IL-1β,Z-D hydrogel can significantly promote the polarization of macrophages towards M2 and highly express TGF-β. All of which are closely associated with the NF-κB signaling pathway [[55], [56], [57]]. The experimental data suggest that the Z-D hydrogel may improve the therapeutic potential of TDSCs in tissue repair by suppressing NF-κB pathway-mediated inflammatory responses. Although various anti-inflammatory agents have been explored for AT treatment, their clinical application has been limited by prevalent issues of tissue adhesion and adverse reactions [58]. In contrast, animal experiments in this study revealed that Z-D hydrogel implantation not only restored mechanical properties in rat AT(maximum tensile strength recovery rate >80 %) but also avoided significant tissue adhesion, highlighting its clinical translational potential.

Further morphological and molecular analyses provided critical mechanistic insights, real-time SMI ultrasound and mechanical testing confirmed that the Z-D hydrogel promoted collagen fiber alignment and biomechanical functional restoration. Immunohistochemical results demonstrated upregulated expression of COL I (mature collagen) and reduced proportion of COL III (scar collagen) in the Z-D group, indicating a shift toward physiological extracellular matrix remodeling. Time-dependent marker analysis revealed early activation of SCX and late-stage upregulation of TNMD, suggesting that the Z-D hydrogel may promote functional tendon regeneration through stage-specific regulation of TDSCs fate. Collectively, these findings support the following mechanistic hypothesis, Immunofluorescence showed that in the early Z-D group, the expressions of IL-6, TNF-α and CD86 were significantly down-regulated and the expression of CD206 was up-regulated, The Z-D hydrogel may inhibit and alleviates inflammation via Zn^2+^-mediated NF-κB pathway inhibition, while synergistically driving TDSCs differentiation toward tendon lineage through biomechanical microenvironment modulation and sustained signaling, ultimately achieving integrated structural-functional repair of injured tendons, this dual regulatory mechanism not only facilitates organized collagen fiber alignment but also substantially improves the biomechanical properties of repaired tissues.

Despite confirming the dual anti-inflammatory and regenerative efficacy of the Z-D hydrogel, several limitations warrant attention, First, the anti-inflammatory mechanism of ZnO NPs remains incompletely elucidated, requiring further clarification of their interactions with specific pathways (NF-κB) through gene knockout or inhibitor experiments. Second, current data derived solely from rat models necessitate subsequent validation in large animal models (rabbits, pigs) to address anatomical, biomechanical, and healing kinetic disparities between rodents and human Achilles tendons.

## 5 Conclusion

This study successfully developed a minimally invasive treatment strategy for AT based on a Z-D hydrogel. Experimental results demonstrated Z-D hydrogel exhibits thermo-sensitive injectability and controlled Zn^2+^ release capability, enabling precise targeting of injury sites; in LPS-induced inflammatory models, The Z-D hydrogel demonstrated a significant suppression of key pro-inflammatory mediators,IL-6 and TNF-α, thereby reversing TDSCs functional inhibition; in rat AT models, Z-D hydrogel implantation effectively restored the biomechanical strength and tissue structure of the Achilles tendon, while promoting functional regeneration through regulation of the collagen I/III ratio and spatiotemporal expression of SCX (early stage) and TNMD (late stage). These result confirm that Z-D hydrogel combines anti-inflammatory and pro-regenerative functions, providing a novel approach for minimally invasive AT treatment. Future research should focus on in-depth elucidation of its molecular mechanisms, validation in large animal models, and optimization of clinical translation pathways.

## CRediT authorship contribution statement

**Xiang Gao:** Writing – review & editing, Writing – original draft, Visualization, Supervision, Resources, Project administration, Methodology, Investigation, Formal analysis. **Senyi Wu:** Writing – original draft, Software, Project administration, Investigation, Formal analysis. **Zheyu Yao:** Software, Data curation. **Yiding Shao:** Resources, Project administration. **Jiahui Feng:** Visualization, Investigation. **Zheyang Yuan:** Supervision, Resources. **Haijiao Mao:** Funding acquisition, Data curation, Conceptualization.

## Declaration of competing interest

The authors declare that they have no known competing financial interests or personal relationships that could have appeared to influence the work reported in this paper.

## Data Availability

Data will be made available on request.
