# Supplementary material for: Engineered decellularized tendon hydrogel with sustained zinc ion release orchestrates anti-inflammatory microenvironment and functional regeneration in Achilles tendinopathy
Source: Mater Today Bio. 2025 Jul 16;34:102104. doi: 10.1016/j.mtbio.2025.102104 (PMC12308006; doi:10.1016/j.mtbio.2025.102104)
Supplement: Multimedia component 1 [file mmc1.docx]

### **1.Scanning electron microscopy of hydrogels and Compressive Modulus of hydrogels**

###
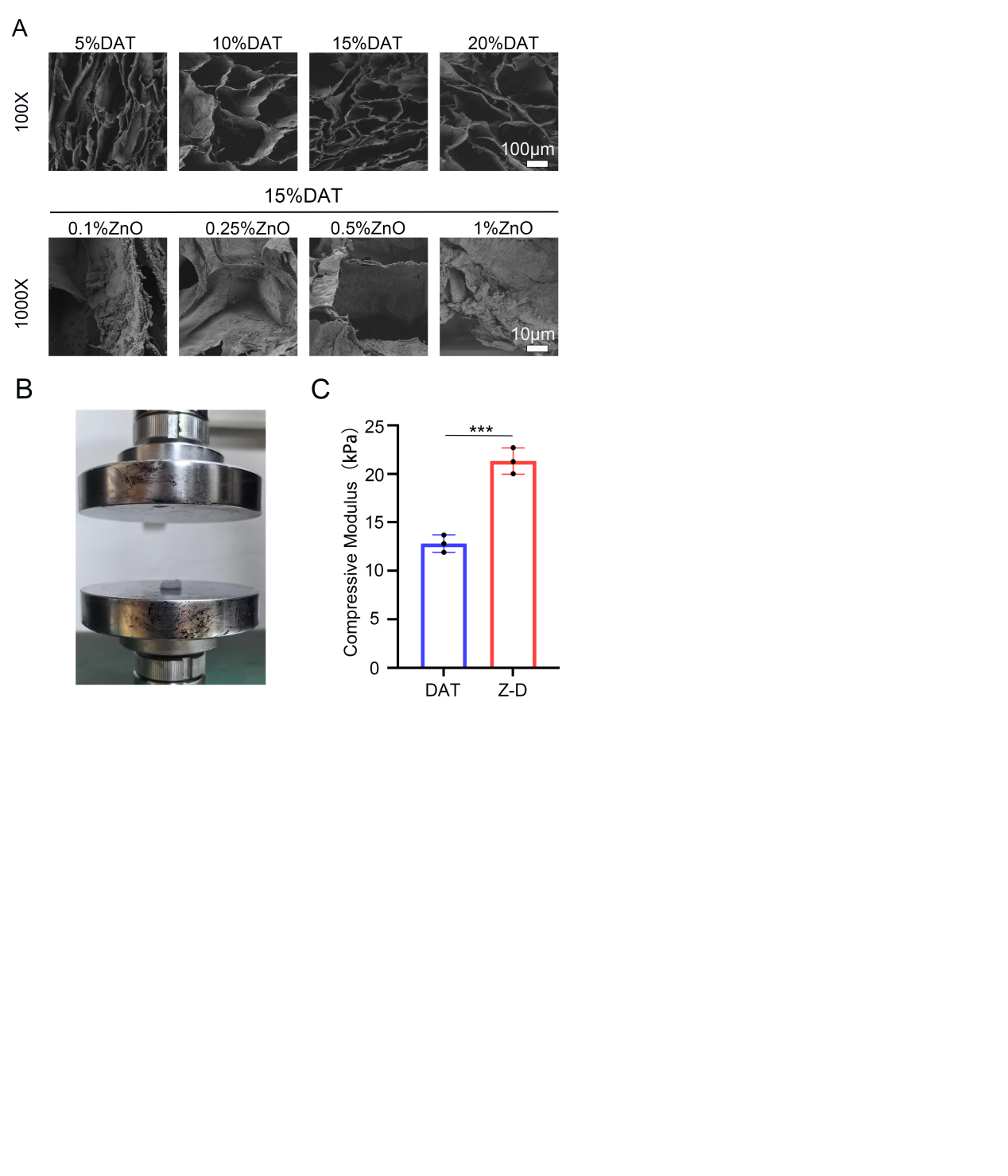


**Supplementary Figure 1**. (A) Scanning electron microscopy (SEM) images of DAT hydrogels with varying concentrations and Z-D hydrogels with different ZnO NPs loadings. SEM images of DAT hydrogels at 5, 10, 15,and 20mg/mL (left to right) under high-magnification views (100×). SEM images of 15 mg/mL DAT hydrogels incorporated with 0.1%, 0.25%, 0.5%, and 1.0% (w/v) ZnO NPs at 10,00× magnification.(B) hydrogels using electromechanical testing system;(C) Quantitative analysis of hydrogels Compressive Modulus (**p* < 0.05, ***p* < 0.01, and ****p* < 0.001).

**2.Immunofluorescence identification of TDSCs**


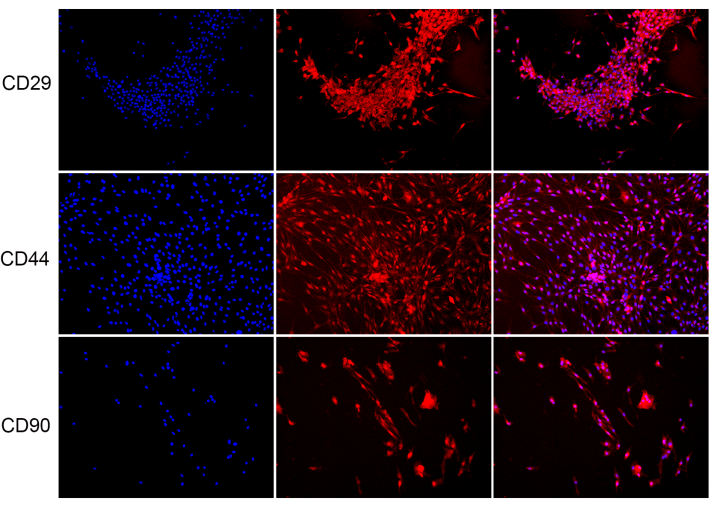


**Supplementary** **Figure 2**.Immunofluorescence identification of TDSCs：CD29,CD44, CD90.

### **3.Statistical analysis of cell migration tests**


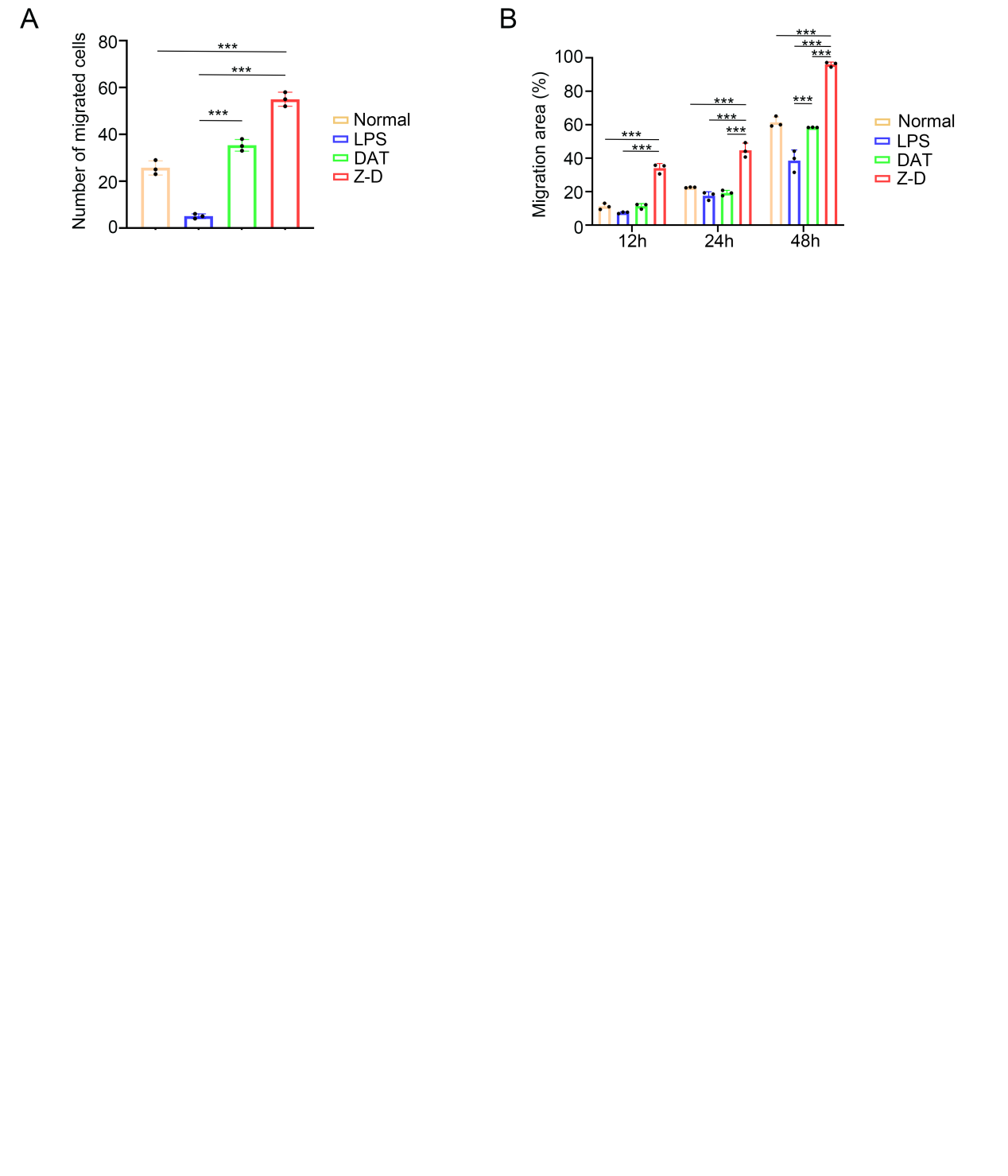


**Supplementary Figure 3.** (A) Quantitative analysis of vertical migration rates in Transwell assays across experimental groups.(B) Quantitative analysis of horizontal migration rates in scratch wound assays. (**p* < 0.05, ***p* < 0.01, and ****p* < 0.001).

**4.Gene Expression Analysis**


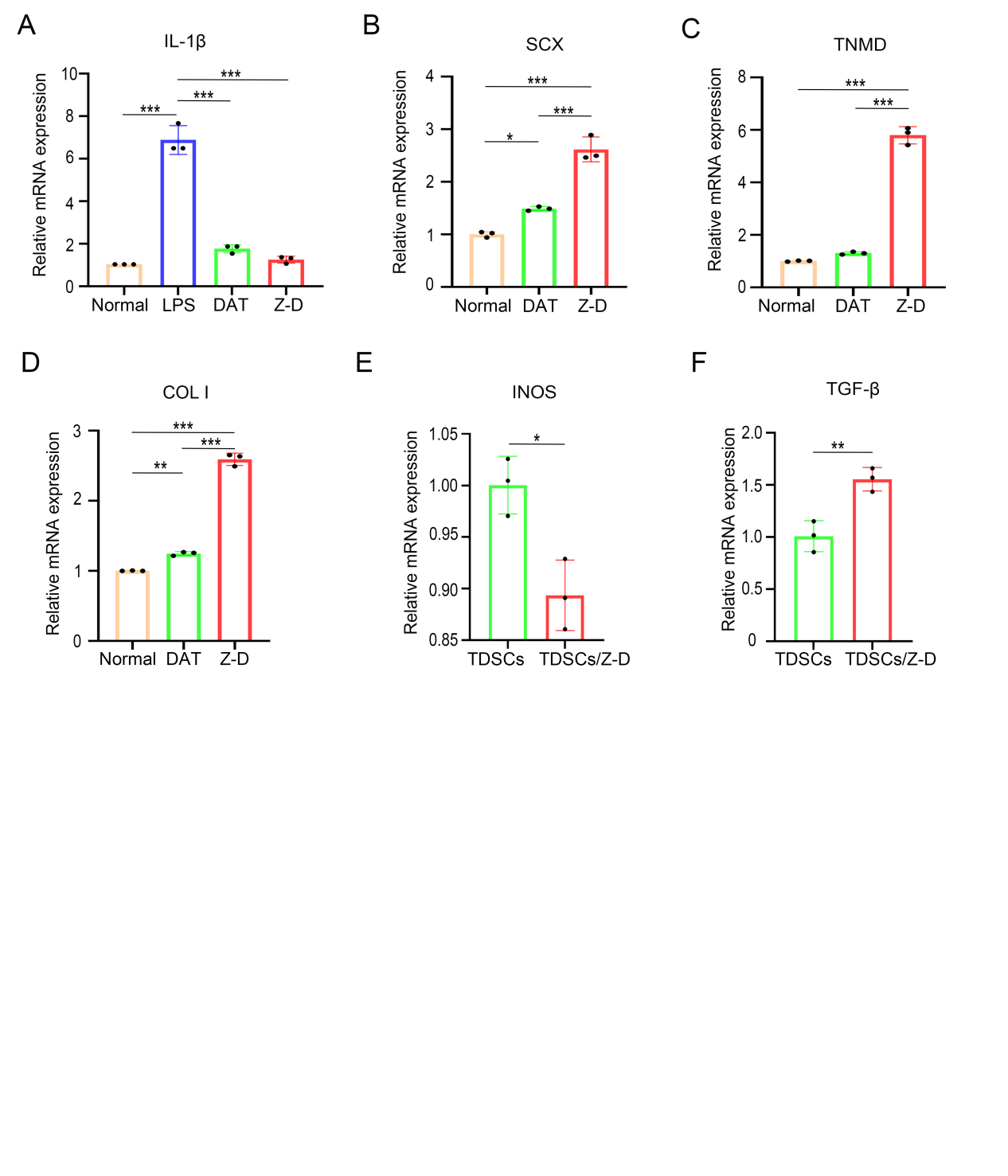


**Supplementary Figure 4.（A）** RT-qPCR analysis of IL-1β gene expression under inflammatory microenvironment conditions,**（B-D）**RT-qPCR analysis of SCX、TNMD and COL1 gene expression **（E-F）**RT-qPCR analysis of INOS and TGF-β gene expression (**p* < 0.05, ***p* < 0.01, and ****p* < 0.001).

**5.Rat Achilles tendinopathy modelling**


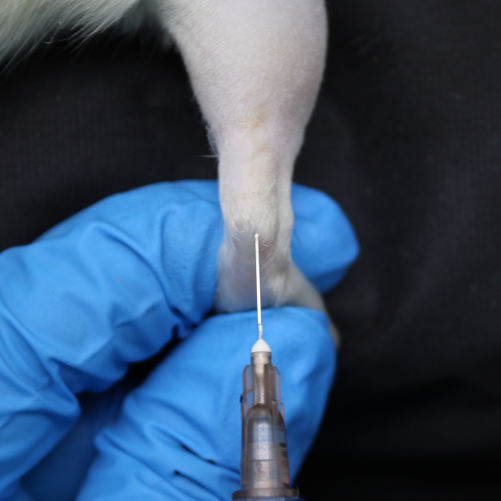


**Supplementary** **Figure 5**. Collagenase was injected into the rat Achilles tendon for modelling.

**6.Histological score and Adhesion score**


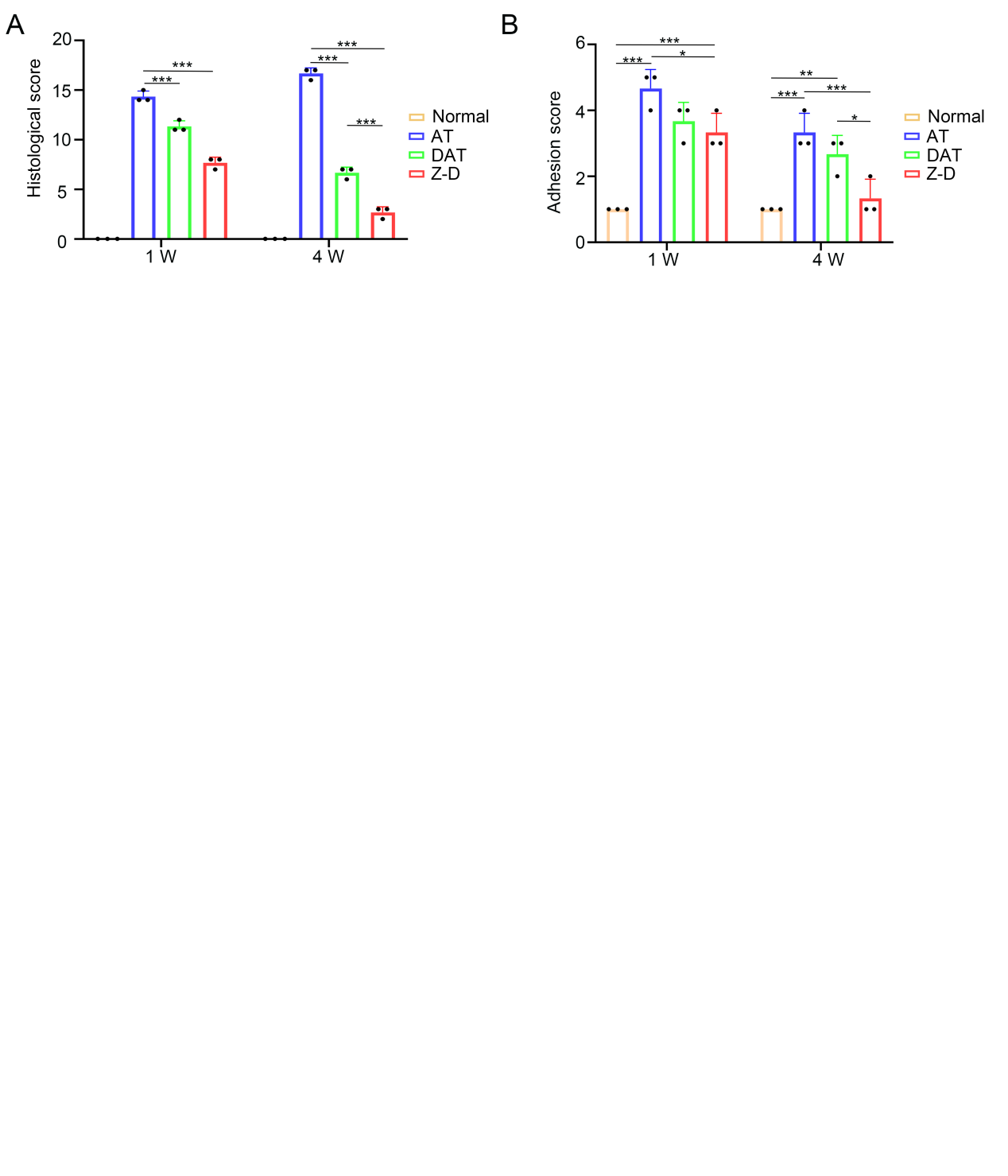


**Supplementary** **Figure 6**. (A) Histological repair scoring based on modified Lane-Sandhu criteria; (B) Quantitative analysis of tissue adhesion extent evaluated using modified Caspers method. (**p* < 0.05, ***p* < 0.01, and ****p* < 0.001).

**7.Immunofluorescence Analysis**


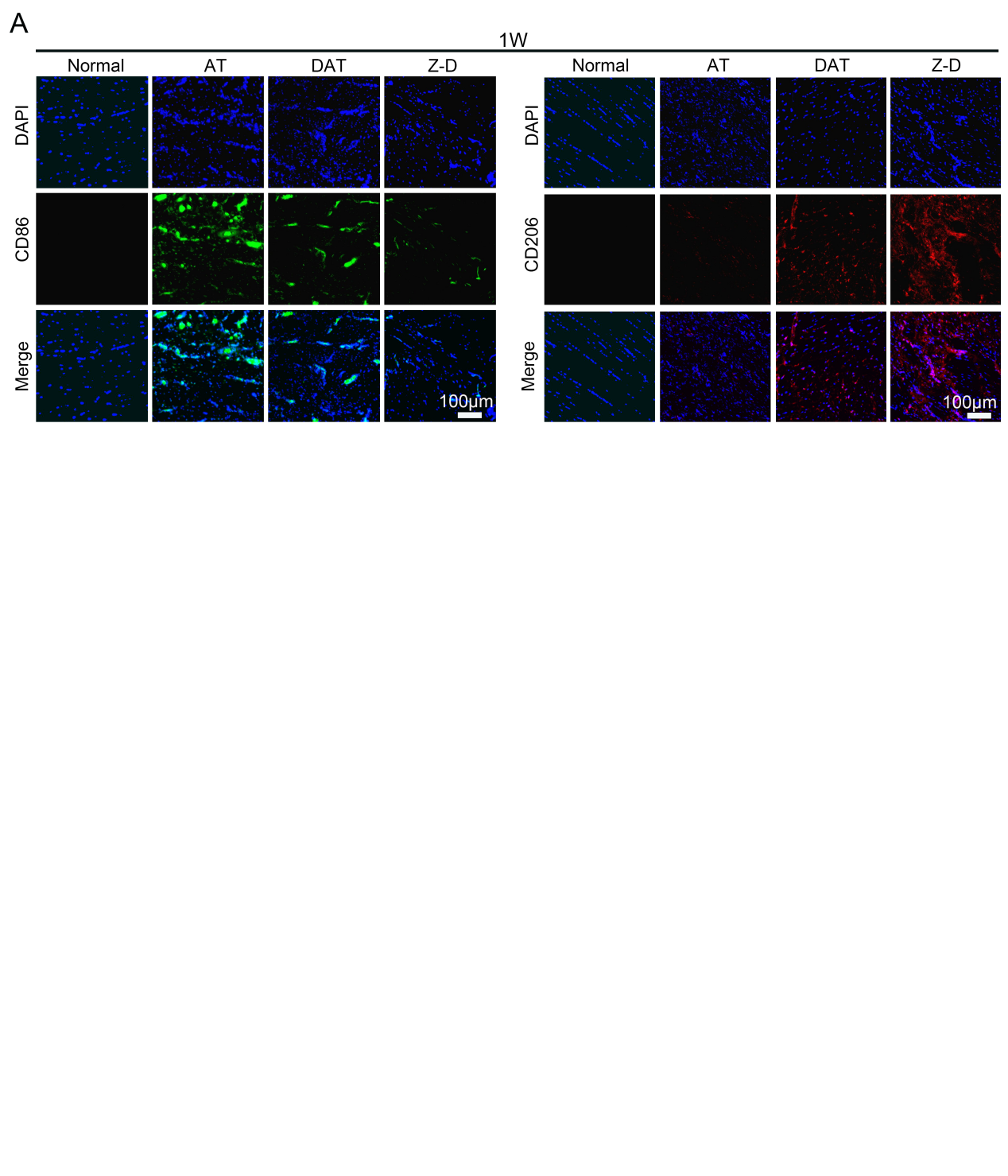


**
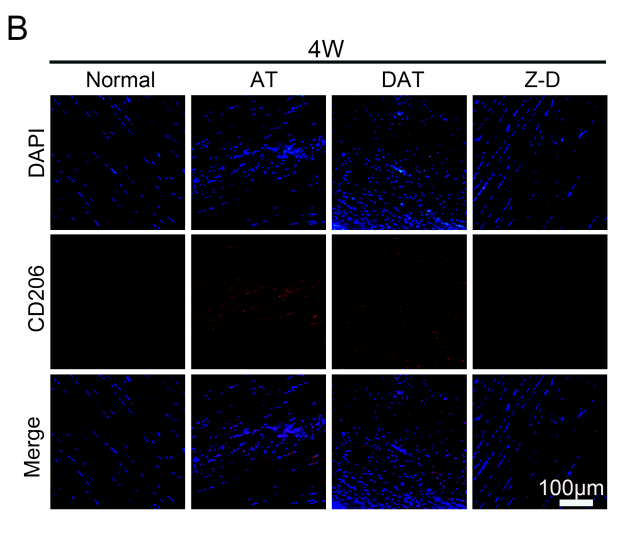
**

**Supplementary** **Figure 7**. (A) CD86 and CD206 immunofluorescence staining in Achilles tendon tissues at 1 weeks post-operation. (B) CD206 immunofluorescence staining in Achilles tendon tissues at 4 weeks post-operation.

**8.*In Vivo* Biosafety Evaluation**


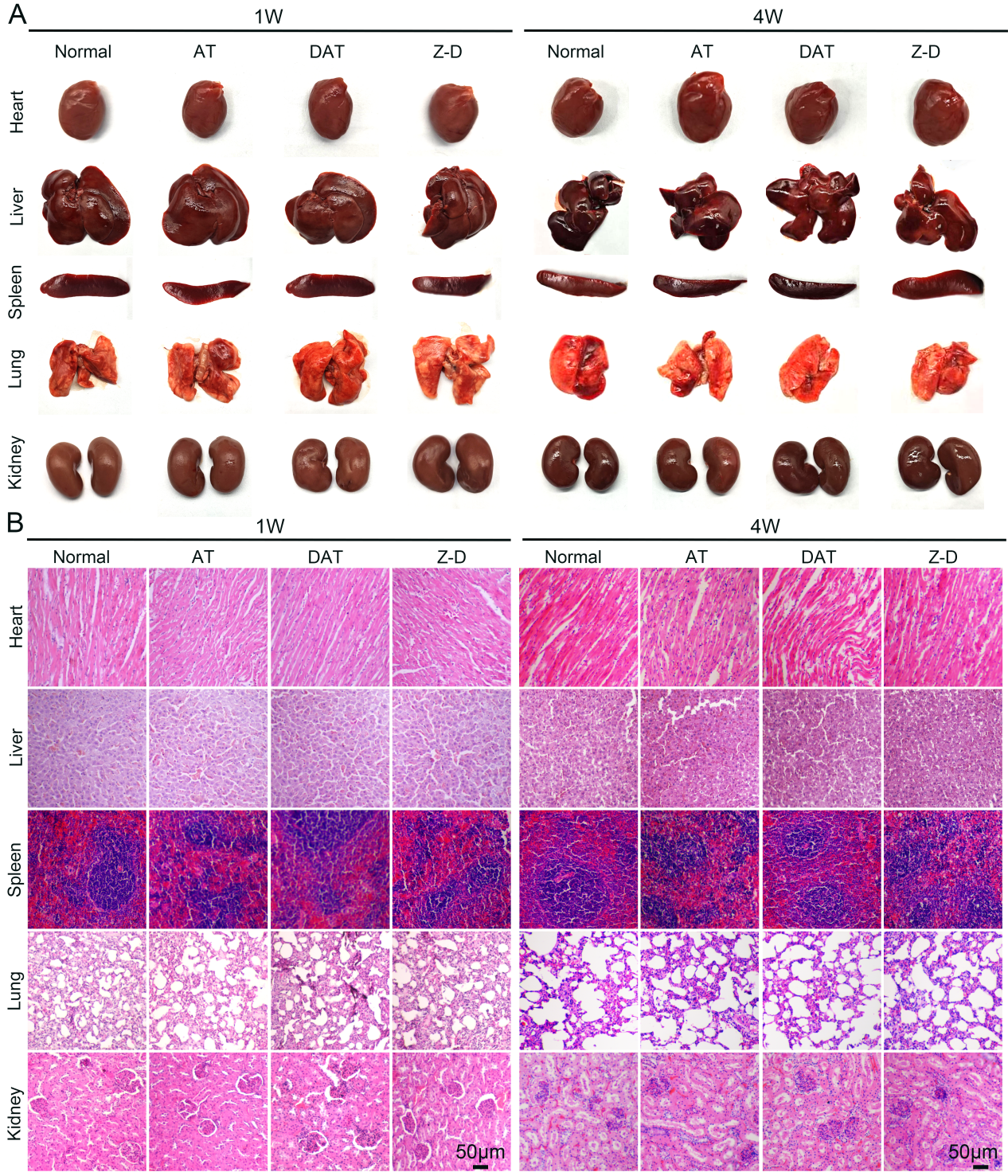


**Supplementary** **Figure 8**. (A-B) Macroscopic images and HE-stained sections of heart, liver, spleen, lung, and kidney tissues from all groups at 1 and 4 weeks post-operation.
